# Supplementary material for: Modelling Lactation Curves for Dairy Sheep in a New Zealand Flock
Source: Animals (Basel). 2023 Jan 19;13(3):349. doi: 10.3390/ani13030349 (PMC9913856; doi:10.3390/ani13030349)
Supplement: Supplementary file 1 [file animals-13-00349-s001.zip › animals-2124017-supplementary.pdf]

Table S1. Least square means (Mean) and standard errors (SE) of regression coefficients for lactation curve of milk (kg), estimated for 130 days after weaning, for different ewe ages, litter size, coat-colour, and days in TAD milking, at Kingsmeade farm, during the production season 2021-2022.

	Milk (kg)									
Effect	a0		a1		a2		a3		a4	
	Mean	SE	Mean	SE	Mean	SE	Mean	SE	Mean	SE
Age										
1	0.493[b]	0.041	-0.28[a]	0.04	0.163[bc]	0.035	-0.078	0.031	0.021	0.033
2	0.657[a]	0.027	-0.427[b]	0.026	0.241[a]	0.023	-0.1	0.02	0.055	0.021
3	0.706[a]	0.027	-0.447[b]	0.026	0.221[ac]	0.023	-0.06	0.02	0.035	0.022
>4	0.691[a]	0.022	-0.435[b]	0.022	0.217[ac]	0.019	-0.068	0.017	0.044	0.018
Litter size										
1	0.626	0.021	-0.382	0.021	0.198	0.018	-0.071	0.016	0.041	0.017
2	0.648	0.022	-0.412	0.021	0.223	0.018	-0.082	0.016	0.038	0.017
Coat colour										
black	0.632	0.03	-0.411	0.029	0.24	0.025	-0.104	0.023	0.048	0.024
white	0.642	0.014	-0.383	0.014	0.181	0.0123	-0.05	0.011	0.03	0.011
[1]TAD days										
0	0.545[b]	0.024	-0.343[bc]	0.023	0.194	0.02	-0.075	0.018	0.012	0.019
14	0.665[a]	0.03	-0.409[ac]	0.029	0.198	0.025	-0.057	0.023	0.035	0.024
21	0.661[a]	0.03	-0.402[ac]	0.03	0.207	0.026	-0.074	0.023	0.051	0.024
28	0.726[a]	0.032	-0.465[a]	0.031	0.25	0.027	-0.098	0.024	0.072	0.026

[1]TAD days= days in twice-a-day milking. [a, b, c]Least square means with different superscripts, within effect, are significantly different (p<0.05).

Table S2. Least square means (Mean) and standard errors (SE) of regression coefficients for lactation curve of fat (g), estimated for 130 days after weaning, for different ewe ages, litter size, coat-colour, and days in TAD milking, at Kingsmeade farm, during the production season 2021-2022.

	Fat (g)											
Effect	a0		a1		a2		a3		a4		a5	
	Mean	SE	Mean	SE	Mean	SE	Mean	SE	Mean	SE	Mean	SE
Age												
1	31.70^{b}	2.31	-10.40^{ac}	1.86	8.60	1.41	-2.49	1.27	0.55	1.31	-1.16^{b}	1.42
2	38.40^{a}	1.53	-13.80^{bc}	1.23	10.00	0.93	-3.41	0.84	-1.21	0.86	3.79^{a}	0.94
3	40.20^{a}	1.53	-15.00^{b}	1.23	9.18	0.93	-1.74	0.84	-1.30	0.87	2.55^{ac}	0.94
>4	40.40^{a}	1.28	-14.80^{b}	1.03	8.97	0.78	-2.48	0.70	-0.10	0.72	1.73^{ab}	0.78
Litter size												
1	37.70	1.20	-13.10	0.97	8.93	0.73	-2.49	0.66	-0.72	0.68	2.18	0.74
2	37.70	1.20	-13.90	1.00	9.49	0.75	-2.57	0.68	-0.30	0.70	1.26	0.76
Coat colour												
black	36.96	1.70	-12.90	1.37	9.31	1.04	-3.07	0.94	-0.26	0.96	1.97	1.04
white	38.40	0.81	-14.10	0.65	9.11	0.49	-1.99	0.44	-0.77	0.46	1.48	0.49
[1]TAD days												
0	31.56^{b}	1.36	-8.95b	1.09	7.88	0.83	-2.80	0.74	0.21^{ac}	0.76	0.15^{bc}	0.83
14	38.96^{a}	1.71	-14.12a	1.37	8.87	1.04	-1.53	0.93	-2.04^{bc}	0.95	2.88^{a}	1.04
21	40.83^{a}	1.72	-16.52a	1.38	10.35	1.05	-1.96	0.94	-1.38^{ac}	0.96	2.52^{ac}	1.05
28	42.68^{a}	1.82	-16.84a	1.46	10.34	1.11	-3.60	0.99	0.62^{a}	1.01	2.27^{ac}	1.11

[1]TAD days= days in twice-a-day milking. [a, b, c]Least square means with different superscripts, within effect, are significantly different (p<0.05).

Table S3. Least square means (Mean) and standard errors (SE) of regression coefficients for lactation curve of protein (g), estimated for 130 days after weaning, for different ewe ages, litter size, coat colour, and days in TAD milking, at Kingsmeade farm, during the production season 2021-2022.

	Protein (g)											
Effect	a0		a1		a2		a3		a4		a5	
	Mean	SE	Mean	SE	Mean	SE	Mean	SE	Mean	SE	Mean	SE
Age												
1	27.67[b]	1.99	-11.76[a]	1.64	8.76	1.17	-2.75	1.03	1.84	1.14	-0.53	1.23
2	34.57[a]	1.32	-16.06[b]	1.08	9.90	0.77	-2.07	0.68	0.19	0.75	2.78	0.81
3	35.88[a]	1.32	-16.73[b]	1.08	9.02	0.77	-0.73	0.68	-0.10	0.76	2.17	0.81
>4	35.38[a]	1.10	-16.19[b]	0.91	9.05	0.65	-1.22	0.57	0.36	0.63	2.13	0.68
Litter size												
1	32.67	1.04	-14.32	0.85	8.79	0.61	-1.80	0.54	0.58	0.59	1.87	0.64
2	34.08	1.07	-16.06	0.88	9.57	0.63	-1.59	0.55	0.56	0.61	1.39	0.66
Coat colour												
black	33.30	1.42	-15.80	1.21	10.45	0.86	-2.53	0.76	0.25	0.84	2.54	0.90
white	33.45	0.70	-14.57	0.57	7.91	0.41	-0.85	0.36	0.89	0.40	0.73	0.43
[1]TAD days												
0	29.15[c]	1.17	-12.98[b]	0.96	9.67[ac]	0.67	-2.64	0.60	0.51	0.67	1.24	0.72
14	34.36[b]	1.47	-15.09[b]	1.20	8.43[ac]	0.85	-0.88	0.76	0.08	0.84	2.20	0.90
21	34.10[b]	1.48	-14.98[b]	1.21	7.88[bc]	0.85	-0.82	0.76	0.99	0.85	0.86	0.91
28	38.19[a]	1.57	-18.85[a]	1.28	10.47[a]	0.90	-1.88	0.81	0.66	0.90	2.54	0.96

[1]TAD days= days in twice-a-day milking. [a, b, c]Least square means with different superscripts, within effect, are significantly different (p<0.05).

Table S4. Least square means (Mean) and standard errors (SE) of regression coefficients for lactation curve of lactose (g), estimated for 130 days after weaning, for different ewe ages, litter size, coat colour, and days in TAD milking, at Kingsmeade farm, during the production season 2021-2022.

	Lactose (g)									
Effect	a0		a1		a2		a3		a4	
	Mean	SE	Mean	SE	Mean	SE	Mean	SE	Mean	SE
Age										
1	23.49[b]	2.04	-14.65[a]	2.04	7.73	1.79	-4.19	1.56	1.27	1.71
2	31.98[a]	1.35	-22.69[b]	1.35	11.73	1.18	-5.21	1.03	3.13	1.13
3	34.05[a]	1.35	-23.49[b]	1.35	10.75	1.18	-3.17	1.03	1.98	1.13
>4	33.22[a]	1.13	-22.86[b]	1.13	10.47	0.99	-3.77	0.86	2.65	0.94
Litter size										
1	30.14	1.06	-20.18	1.06	9.59	0.93	-3.79	0.81	2.30	0.89
2	31.23	1.09	-21.66	1.09	10.74	0.96	-4.38	0.84	2.21	0.91
Coat colour										
black	30.51	1.50	-21.60	1.50	11.65	1.32	-5.40	1.15	2.59	1.26
white	30.85	0.71	-20.25	0.71	8.69	0.62	-2.78	0.55	1.92	0.60
[1]TAD days										
0	26.34[b]	1.20	-18.57[a]	1.20	9.55	1.05	-4.14	0.19	0.88	1.01
14	32.22[a]	1.50	-21.48[ac]	1.50	9.24	1.32	-2.92	1.15	2.07	1.27
21	31.84[a]	1.52	-20.91[ac]	1.52	9.93	1.33	-3.90	1.16	2.86	1.28
28	34.70[a]	1.60	-24.04[bc]	1.60	12.18	1.40	-5.23	1.23	3.92	1.35

[1]TAD days= days in twice-a-day milking. [a, b, c]Least square means with different superscripts, within effect, are significantly different (p<0.05).
